# Effect of mouthwashes on the force decay of polymeric ligature chains used for dental purposes: a systematic review and meta-analysis

**DOI:** 10.1186/s12903-023-03240-3

**Published:** 2023-08-04

**Authors:** Carolina Andrés Castelló, Natalia Zamora-Martínez, Vanessa Paredes-Gallardo, Beatriz Tarazona-Álvarez

**Affiliations:** Faculty of Medicine and Dentistry, University of Valencia, Valencia, Spain

**Keywords:** Force decay, Orthodontic elastomeric chain, Mouthwashes, Mouth rinse, Alcohol, Chlorhexidine, Sodium fluoride, Persica, Bleaching agent

## Abstract

**Background:**

External factors such as the daily use of antimicrobial mouthwashes to maintain oral hygiene and to reduce the microbial activity can contribute to alter the mechanical properties of the elastomeric chains used during orthodontic treatments, causing loss of effectiveness. This systematic review and a meta-analysis assessed the rate of force decay and degradation of the polymeric chains depending on the type of mouthwash.

**Methods:**

A systematic search of the literature were there was an exposure of orthodontic elastomeric chains to certain mouthwashes was conducted in the electronic databases of PubMed, Cochrane Library (CENTRAL), Scopus, EMBASE and Web of Science, as well as grey literature (Opengrey). No limit was placed on publication year and research was done up to June 2022. Based on inclusion/ exclusion criteria, data were extracted by two independent reviewers. For the quantitative analysis, studies were analysed with a mixed-effect (random effect) meta-regression model, with beta coefficients and R [2] values. I [2] index and Q and Egger tests were used to find heterogeneity among studies.

**Results:**

A total of 178 potentially eligible studies were identified, of which 14 were eventually included in the qualitative analysis and 14 in the quantitative meta-analysis. The meta-analysis showed that all the mouthwashes were associated with a greater force decay than the control groups. After 7 days (p = 0.005) significant differences were found among the different mouthwashes, with those containing alcohol having significantly higher impact on the force decay than those containing chlorhexidine 0.2%, sodium fluoride or Persica. However, at 24 h (p = 0.200), 14 days (p = 0.076), 21 days (p = 0.120) and 28 days (p = 0.778) no statistically significant differences among the different mouthwashes were found, although those containing alcohol presented a strong tendency.

**Conclusion:**

Although mouthwashes tend to increase the speed of force decay of elastomeric chains, especially those containing alcohol, clorhexidine 0.2% can be a good alternative due to its low impact on the force decay and its ability to maintain low microbial activity. More in vitro and in vivo studies comparing different manufacturers and other agents should be performed.

**Supplementary Information:**

The online version contains supplementary material available at 10.1186/s12903-023-03240-3.

## Background

Orthodontic treatments are clinical procedures carried out by the dentists to relocate the misaligned teeth. Arch wires, brackets, screws, ligature ties, elastic bands and chains are the most common orthodontic appliances to induce tooth movement. Specifically, elastomeric chains are polyurethane elastomers frequently used in multiple orthodontic treatments to correct small rotations or helping with the closure of small distances between teeth [1, 2].

Despite the widespread use of elastomeric chains, the speed of degradation of the mechanical properties and loss of strength upon activation causes their effectiveness to decrease exponentially [2]. The strength of polyurethane elastomers decays with time and the slump rate increases with hydrolysis. Andreassen and Bishara, in 1970, already demonstrated that 55% of the loss of strength occurred one hour after the initial degradation. Already in these first studies and published results, the loss of the mechanical properties of these elements became a significant clinical problem of interest to researchers [2].

In fact, many other studies [3, 4] have reported that the elastomeric chains suffer a loss of strength between 50% and 70% at 24 h, followed by a more constant phase with a 10–20% of force decay during the following 4 weeks [1]. Others, have reported similar losses within the first week of use [3, 5].

On the other hand, certain external factors, such as oral cavity temperature, the intraoral environment, the action of substances that are contained in the saliva, food or beverages, the changes in the salivary pH or the exposure to ultraviolet light are reported to participate in the degradation of the elastomeric chains [6–13].

Other factors such as the poor oral hygiene or the usage of mouthwashes also play an important role. Particularly, the installation of orthodontic appliances could lead to the accumulation of microorganisms within the oral cavity, such as Streptococcus mutans, Lactobacilli, or Candida, producing acidic compounds which can cause tooth decay and demineralization during orthodontic treatments [5–7]. Antimicrobial mouthwashes are often used to mitigate this issue. Recently, there are published guidelines advising the use of some mouthrinses before dental appointments, such as cetylpyridinium chloride, clohexidine gluconate or hydrogen pexoxide [6], but it appears that its use not only reduces the presence of microorganisms, but also alters the properties of orthodontic appliances. [7].

Specifically, the repeated use of mouthwashes and certain agents found in them [8, 11, 13–15, 19] as a reinforcement measure in oral hygiene during orthodontic treatment can have repercussions involving the degradation of the strength of elastomeric chains. It is known that these agents play a very active role in altering the physical properties of these devices, which ultimately results in their loss of effectiveness [7, 8]. Some authors [5, 11] reported strength degradation percentages of 71.6% and 49% within 28 days when mouthwashes such as Listerine® [11] and chlorhexidine rinses were used, respectively. Other agents, such as alcohol [8] or bleaching agents [7], also increased the rate of degradation of the strength of these materials by up to 86.45%.

Due to the great variety of antimicrobial mouthwashes prescribed as an adjuvant in orthodontic treatments, the objective of this systematic review and a meta-analysis was to investigate the influence of the components of the most frequently used mouthwashes on the degradation of the mechanical properties of the elastomeric chains.

## Method

### Registration and focused question

This systematic review was conducted following the Preferred Reporting Items for Systematic Reviews and Meta-Analyses (PRISMA) statement and the Cochrane manual for systematic reviews (version 6.0; Higgins JPT 2019) [9]. The systematic review protocol was previously registered in Open Science Framework (OSF) Registries (DOI: 10.17605/OSF.IO/V7NYB).

The focused PICO research question was: to what extent the use of antimicrobial mouthwashes affects the degradation of the mechanical properties and strength of the elastomeric chains used in orthodontic treatments?

### Eligibility criteria

The following eligibility criteria were applied: 1) Population: Controlled clinical trials, randomized clinical trials (RCTs), cohort studies, case–control studies, cross-sectional studies, multicentre studies and in vitro studies conducted on elastomeric chains for orthodontic purrposes. 3) Interventions: Application of any mouthwash to the elastomeric chain during a certain period of time. 4) Outcome measure: assessment of force decay in elastomeric chains in relation to mouthwash and time of exposure.

### Search strategy and information sources

A comprehensive literature search was performed using the following databases: PubMed, Cochrane Library (CENTRAL), Scopus, EMBASE, and Web of Science. An electronic grey literature search was performed using Opengrey. If necessary, the authors of the articles were contacted by email to request missing information. The reference lists of included studies were manually searched to identify and screen articles not found in databases that might meet the inclusion criteria. The search was conducted up to June 2022 and sought to identify all articles related to the topic that have been published up to that date, with no constraints in terms of language. The search strategy included a combination of medical subject heading (MESH) terms (mouth rinses [MeSH Terms]) and free text words for PubMed and was optimized for each database. Boolean operators (“OR” and “AND”) were used to join terms (MeSH/non-MeSH) related to the research question. These keywords were divided into four groups: 5 keywords related to elastomeric chains, 2 secondary keywords related to strength degradation, 11 keywords related to speed or speed of tooth movement, and 9 keywords related to buccal rinses and their components. Searches for all possible combinations of terms in the groups were conducted (Table 1). Identified articles were exported to Refworks ProQuest software for the removal of duplicates.

**Table 1:** Search strategy for identifying studies in primary electronic databases

Search equation:
*((((orthodontic chain) OR (elastomeric chain) OR (elastic chain) OR (power chain) OR (chain))) AND (((force decay) OR (force degradation)))) AND (((mouthwashes [MeSH Terms]) OR (mouthwash) OR (mouth rinse) OR (mouthrinse) OR (mouth-rinse) (alcohol) OR (chlorhexidine) OR (fluorure) OR (bleaching)))*
*((‘orthodontic’/exp OR orthodontic) AND chain OR ((‘elastomeric’/exp OR elastomeric) AND chain) OR (elastic AND chain) OR ((‘power’/exp OR power) AND chain) OR chain) AND ((‘force’/exp OR force) AND decay OR ((‘force’/exp OR force) AND (‘degradation’/exp OR degradation))) AND (‘mouthwashes’/exp OR mouthwashes OR ‘mouthwash’/exp OR mouthwash OR ((‘mouth’/exp OR mouth) AND rinse) OR ‘mouthrinse’/exp OR mouthrinse OR ‘mouth rinse’/exp OR ‘mouth rinse’ OR ‘alcohol’/exp OR alcohol OR ‘chlorhexidine’/exp OR chlorhexidine OR fluorure OR ‘bleaching’/exp OR bleaching)*

### Screening process and data collection

Two authors (CA and NZ) systematically screened all the titles and abstracts of all identified articles independently. If disagreement occurred, a third author (BT) was consulted. If the abstract did not contain enough information to include or exclude a particular article, the authors read the full article before making a final decision. Once potential studies were identified for inclusion, both authors retrieved and reviewed the full texts of the articles (CA and NZ). General information was extracted from the selected studies, including year of publication, type of elastomeric chain, number of links, commercial company, measurement intervals, distance between pins, type of rinse or agent studied, and controls. Subsequently, the full texts of all the articles were read, and the reasons for rejection of the excluded articles were recorded (Supplementary Table 1).

### Risk of bias and quality assessment of individual studies

The quality of the included studies and the risk of bias were assessed by the same investigators, who worked independently. To do this, a protocol adapted from the QUIN tool developed by Sheth et al. [10] was used, based on the following parameters: clearly stated aims, explanation of sample size calculation, presence of a control group well defined, operator blinding, number of links used, distance between pins and statistical analysis. Researchers scored each of the criteria as adequately specified = 2 points, inadequately specified = 1 point, not specified = 0 point, and not applicable = exclude criteria from calculation. The scores were then added to obtain a total score for a particular in vitro study. The scores thus obtained were used to grade the in vitro study as high, medium, or low risk (> 70%=low risk of bias, 50–70%=medium risk of bias, and < 50% = high risk of bias) by using the following formula: Final score = Total score×100/ 2×number of criteria applicable).

Any disagreement between the researchers was resolved by consensus, and if there were any concerns, a third researcher (BT) was consulted.

Cohen’s kappa coefficient was used to assess the consistency of the intra- and interexaminer evaluations of risk of bias; a Cohen’s kappa coefficient of 0.87 was considered to indicate high reproducibility.

The protocol for this review also included a visual inspection of an improved Funnel plot and Egger’s test for statistical assessment of bias.

The authors have also used the GRADE approach software (GRADEpro GDT: GRADEpro Guideline Development Tool [Software]. McMaster University, 2020 (developed by Evidence Prime, Inc.). Available from gradepro.org.) to grade the available evidence.

### Statistical analysis and sensitive analysis

For each of the articles and for each type of mouthwash, the standardized mean difference (SMD) between the mouthwash and its control group, with the corresponding sample variance, was obtained, thus representing the advantage of the mouthwash over the control treatment.

To compare each mouthwash with respect to the control treatment, a meta-analysis was performed using random-effects models and a maximum likelihood estimator with a z distribution test and 95% confidence intervals (95% CIs).

To compare the force retention associated with the mouthwashes, a meta-regression model was estimated; the moderating variable was the type of mouthwash and under the random-effects approach (mixed effects). Finally, a new meta-regression model including all the mouthwashes assessed by the authors evaluated the relevance of the type of connector.

For the assessment of heterogeneity, the I [2] index and the corresponding test statistic of nullity of Q were calculated. A Galbraith plot was generated to explore heterogeneity. The level of significance used in the analyses was 5% (α = 0.05).

The software used to perform the meta-analysis was R 3.5.1 (R Core Team (2013). R: A language and environment for statistical computing. R Foundation for Statistical Computing, Vienna, Austria. URL (http://www.R-project.org/).

## Results

### Selection of studies

The search identified 178 preliminary references, of which 36 were found in PubMed, 46 in Scopus, 1 in the Cochrane Library, 74 in Web of Science, 20 in EMBASE, 0 in the grey literature and 1 in the manual search of cited references. After excluding 30 duplicates, the remaining 148 were examined. Of these, 123 were excluded after reading the title and abstract, as they were not related to the research question.

**Fig. 1:**
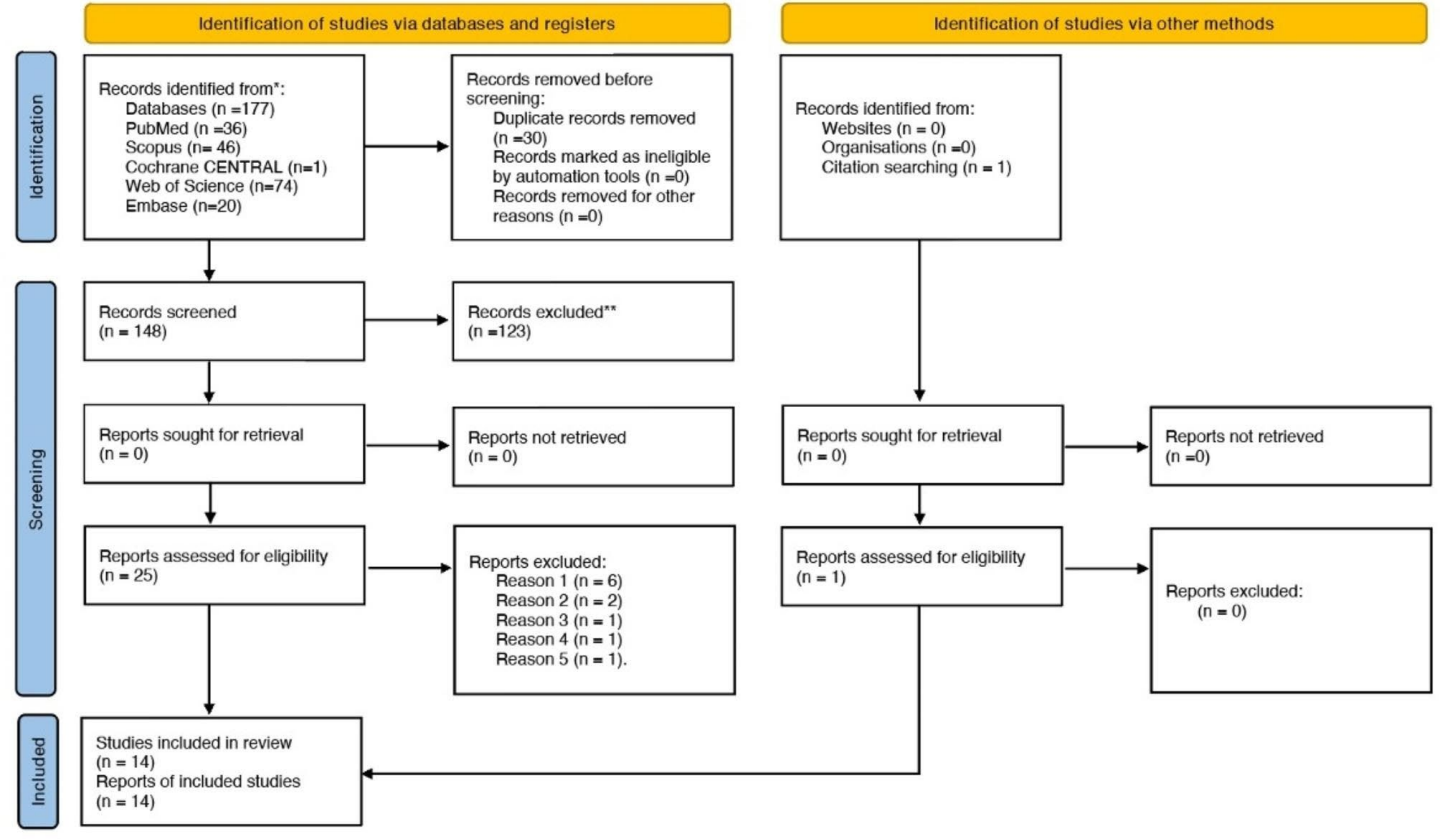
PRISMA 2020 flow diagram for new systematic reviews which included searches of databases, registers and other sources

After reviewing the full texts of the remaining 25 articles, 11 were excluded because they did not meet the inclusion criteria. Finally, 14 articles met the inclusion criteria and were selected for qualitative analysis. The same articles were included in the quanitative meta-analysis. The PRISMA 2020 flowchart (Fig. 1) provides an overview of the article selection process.

### Characteristics of the studies

Regarding sample size, three studies showed much higher sample values than those in other studies: Menon et al. [11], had a sample of 840 elastomeric chains; Kumar et al. [12], 480 elastomeric chains; and Larrabee et al. [8], 450 elastomeric chains. On the other hand, in the studies of Al-Ani [13], Sadeghian et al. [14] and Pithon et al. [7, 15], the samples barely reached the 80 modules. Regarding the type of links used in the studies, all the studies analysed short-link chains. In the study of Omidkhoda et al. [16], closed-link chains in addition to short-link chains were analysed.

The number of links used to measure the strength of the elastomeric chains was quite different. Behnaz et al. [17] used elastomeric chains with 6 links. Authors such as Sadeghian et al. [14], Javanmardi and Salehi [4], Odmikhoda et al. [16] and Pithon et al. [7, 15] used 5 links to carry out their measurements. Oshagh et al. [18] and Kumar et al. [12] used 4 links. Menon et al. [11], Ramachandraiah et al. [19] and Larrabee et al. [8] used only 3-link sections. In the studies by Al-Ani [13] and Nahidh et al. [20], the number of links used for force measurement was not specified.

Regarding the commercial manufacturers of the elastomeric chains, many differences were found between studies. Morelli® elastomeric chains were used in the Al-Ani [13] and Pithon et al. [7, 15] studies. 3 M® brand chains were used in the Ramachandraiah et al. [19] and Kumar et al. [12] studies. Rocky Mountain® chains were selected in the study by Larrabee et al. [8] Ortho Technology® chains were used by Oshagh et al. [18] and Nahidh et al. [20]. American Orthodontics® chains were used in the Benhaz et al. [17], Mirhashemi et al. [4] and Sadeghian et al. [14]. Omidkhoda et al. [16] used elastomeric chains of Dentaurum®, and Menon et al. [11] used chains of Ortho Organizers®. In general, all the studies used chains from a single commercial company, except Ramachadraiah et al. [19], who used chains from three different commercial companies: Ortho Plus®, 3 M® and Ortho Organizers®.

In relation to the distance between the pins to which the elastomeric chains were attached to measure the force during the measurement intervals there were also discrepancies between authors. Menon et al. [11], Pithon et al. [7, 15] and Larrabee et al. [8] used a distance between the holding pins of 23.5 mm. Behnaz et al. [17], Sadeghian et al. [14], Omidkhoda et al. [16], Kumar et al. and Oshagh et al. [12, 18] used pins with a distance of 25 mm. Al-Ani [13] and Nahidh et al. [20] used a distance of 29 mm, Javanmardi and Salehi [4] used a distance of 15 mm, and Ramachandraiah et al. [19] used distances of 18 and 22 mm for prestretched and unstretched chains, respectively.

The intervals applied to assess the strength degradation of the selected elastomeric chains were quite similar among studies. All the authors measured strength degradation at 24 h, 7 days, 14 days, 21 days, and 28 days. Only in the study by Al-Ani [13] was the force not recorded at 14 and 28 days.

Of the mouthwashes and agents used in the studies included in this review, the most frequently studied one was Listerine® (26.9% alcohol) [11–15, 19]. Some authors studied other types of Listerine®-brand mouthwashes, such as Cepacol®, Listerine® Zero (0% alcohol) [15], Listerine® Total Care Zero (NaF 0.02%) [15], Listerine® Healthy White (NaF 0.02% + hydrogen peroxide) [17], Listerine® Whitening (hydrogen peroxide) [15] and Listerine® Green Tea [20]. Rinses containing chlorhexidine 0.2%, such as Chlorhexidine Plus® [11], Cordosyl® [18] and Cleanform®, and chlorhexidine 0.12%, such as Periogard®, were also studied. Other authors reported the results of sodium fluoride (NaF)-based mouthwashes, such as Orthokin® (NaF + chlorhexidine) [4], Sensikin® (NaF + potassium nitrate) [4], Oral B® 0.05% (NaF 0.05%) [18] and Colgate® Phos-Flur (NaF 0.04%) [11]. Plax mouthwashes (CPC 0.075% + NaF 0.05%) and Plax Whitening (CPC 0.05% + 1.5% hydrogen peroxide) were also studied [7]. Finally, Nahid et al. [20] studied herbal mouthwashes, such as Tebodont, Aloe-dent and Silca Herb [20], while several authors included Persica-based mouthwash in their studies. [4, 5, 16].

It should be noted that various authors focused solely on the study of specific agents, such as alcohol at different concentrations, frequently found in a wide variety of mouthwashes. The concentrations studied were 26.9%8, [11], 21.6% [19], 14% [8] and 8.38% of alcohol. [19].

Agents such as chlorhexidine and sodium fluoride have also been extensively studied independently. Chlorhexidine was studied at a concentration of 0.2% in most relevant studies [5, 11, 16], but it was also studied at a concentration of 0.12% in the study by Pithon et al. [15]. The included studies that analysed the results obtained by NaF did so at concentrations of 0.4% [11], 0.2% [5] and 0.05% [14, 16].

In the control groups, the agents used were distilled water and artificial saliva. Of the 14 articles selected, half used distilled water [7, 8, 12, 13, 15, 18, 20], and the other half used artificial saliva. [4, 5, 11, 14, 16, 17, 19].

All the studies applied the mouthwash for 60 s twice a day, except for the studies by Pithon et al. [7, 15] and Behnaz et al. [17], who applied the mouthwash for 30 s twice a day.

The characteristics of the studies, as well as the data extracted from the articles included in this review, are summarized in Table 2.

**Table 2:** Characteristics of the included studies. M: Morelli; OO: Ortho Organizers; AO: American Orthodontics; OT: Ortho Technology (USA); 3 M: 3 M Unitek; OP: Ortho Plus; D: Dentaurum (Alemania); RMO: Rocky Mountain; P: pre-stretched; SP: without pre-stretched

AUTHOR	STUDY DESIGN	SAMPLE	CHAIN	LINKS	MANUFACTURER	DISTANCE BETWEEN PINS	TIME	TYPE OF MOUTHWASH/AGENT	CONTROL GROUP	MOUTHWASH APPLICATION	RESULTS	QUALITY
**Al – Ani** [13]	In vitro	72	short	-	M	29 mm	0 h, 24 h, 7d, 21d	Listerine®- Zero (without alcohol) Listerine® Original (26,9% alcohol)	Distilled water	60 seg / 12 h	All groups with alcohol showed an increase in strength loss compared to the non-alcoholic and distilled water control group (p < 0.05).	**MEDIUM**
**Menon et al.** [11]	In vitro	840	short	3	OO	23,5 mm	0 h, 24 h, 7, 14, 21, 28 d.	Listerine®; Colgate Phos Flur®, Clorhex Plus®; 26,9% alcohol; 0.04% NaF; Clorhexidine 0,2%	Artificial saliva	60 seg / 12 h	At 24 h, the greatest loss of strength (49.48%) was observed in the 26.9% Alcohol group in the short elastomeric chains. The best result was obtained by 0.2% chlorhexidine with a loss of 46.7% (p < 0.5)	**LOW**
**Behnaz et al.** [17]	In vitro	160	short	6	AO	25 mm	0 h, 24 h, 7, 14, 21, 28 d.	Listerine® Total Care Zero Listerine® Healthy White	Artificial saliva	30 seg / 12 h	At 24 h the greatest loss of decomposition of the elastomeric chains was produced in all the groups. In the control group, the loss of strength was 42.18%, while in daily mouth rinses with whitening agents it was 48.34% (Listerine ® Total Care Zero) and 53.38% (Listerine ® Healthy White) respectively (p < 0.05)	**LOW**
**Nahidh et al.** [20]	In vitro	440	short	-	OT	29 mm	0 h, 24 h, 7, 14, 21, 28 d.	Listerine® green tea. (Camellia Sinensis Green tea; NaF) Tebodont (Melaleuca Alternifolia, tea tree oil) Aloe - dent (aloe vera, peppermint oil, mentol, Melaleuca Alternifolia) Herba Silca (Milenrama, chamomile, calendula, salvia)	Distilled water	60 seg / 12 h	They found the greatest degradation of strength in the control group at 24 h of 52%. There were no statistically significant differences with the herbal rinses (p < 0.05)	**LOW**
**Ramachandraiah et al.** [19]	In vitro	180	corta	3	3 M; OP; OO	18 mm (P). 22 mm (SP).	0 h, 24 h, 7, 14, 21, 28 d.	Listerine®; Wokadine; Alcohol 21,6%; Alcohol 8,38%	Artificial saliva	60 seg / 12 h	In all groups, including the control group, the greatest loss of strength was obtained at 24 h in the group of elastomeric chains without pre-stretching compared to the pre-stretched chains that obtained better results.	**LOW**
**Mirhashemi et al.** [4]	In vitro	315	Sin enlace	-	AO	24 mm	0 h, 24 h, 7, 14, 21, 28 d.	Persica; Clorhexidine 0,2% Sodium Fluorure 0,2%; CLX + Sodium Fluorure	Artificial saliva	60 seg / 12 h	A loss of strength was observed with statistically significant results for all groups (p < 0.001). The greatest strength degradation occurred at 24 h in the CLX + NaF group.	**MEDIUM**
**Sadeghian et al.** [14]	In vitro	90	short	5	AO; D	25 mm	0 h, 24 h, 7, 14, 21, 28 d.	Sodium Fluorure 0,05%; Listerine®	Artificial saliva	60 seg / 24 h	The results showed that the effect of the commercial house on the strength of the elastomeric chains was statistically significant and that the average strength of the AO chains was higher than that of the Dentaurum house (p = 0.001). Both NaF and Listerine ® intensified the loss of strength of the elastomeric chains (p < 0.001)	**LOW**
**Javanmardi and Salehi** [3]	In vitro	80	short	5	OT	15 mm	0 h, 24 h, 7, 14, 21, 28 d.	Orthokin®; Sensikin®; Persica	Artificial saliva	60 seg / 12 h	The highest percentage of strength degradation was in the Persica group. The Orthokin® mouthwash was the one that obtained the best results compared to the other groups, including the control group.	**LOW**
**Omidkhoda et al.** [16]	In vitro	120	short/ closed	5/7	D	25 mm	0 h, 24 h, 7, 14, 21, 28 d.	Clorhexidine 0,2%; Persica Sodio Fluorure 0,05%	Artificial saliva	60 seg / 24 h	Chlorhexidine caused the greatest loss of strength at 24 h (27.24% in closed connectors) followed by NaF and artificial saliva. Persica obtained the best results.	**LOW**
**Oshagh et al.** [18]	In vitro	-	closed	4	OT	25 mm	0 h, 24 h, 7, 14, 21, 28 d.	Sodium Fluorure 0,0% Clorhexidine 0,2%; Té	Distilled water	60 seg / 24 h	The greatest loss of strength was observed at 24 h in all groups, but there were no statistically significant differences in terms of strength degradation with the different mouthwashes (p < 0.05).	**LOW**
**Kumar et al.** [12]	In vitro	480	short	4	3 M	25 mm	0 h, 24 h, 7, 14, 21, 28 d.	Coca-Cola; Listerine® Tea	Distilled water	60 seg / 12 h	Tea caused the greatest degradation of strength followed by the Listerine ® and Coca-Cola group compared to the control group with statistically significant results (p < 0.01)	**LOW**
**Pithon et al.** [15]	In vitro	90	short	5	M	23,5 mm	0 h, 24 h, 7, 14, 21, 28 d.	Clorhexidine 0,12%; Clorhexidine 0,2% Periogard® 0,12%; Cleanform®	Distilled water	30 seg / 12 h	Chlorhexidine did not show a statistically significant influence on the pattern of degradation of the strength of the elastomeric chains (p < 0.05).	**LOW**
**Pithon et al.** [7]	In vitro	108	short	5	M	23,5 mm	0 h, 24 h, 7, 14, 21, 28 d.	Plax; Listerine® Plax Whitening; Listerine® Whitening	Distilled water	60 seg / 12 h	All groups obtained the greatest loss of strength at 24 h. After 28 days, the greatest loss of strength occurred in the control group (artificial saliva). The presence of bleaching agent had no relevance in the results obtained.	**LOW**
**Larrabee et al.** [8]	In vitro	450	short	3	RMO	23,5 mm	0 h, 24 h, 7, 14, 21, 28 d.	Alcohol 14%; Alcohol 26,9% Cepacol® (alcohol 14%); Listerine® (alcohol 26,9%)	Distilled water	60 seg / 12 h	Cepacol® mouthwash obtained 54.2% loss of strength at 24 h compared to 53% for Listerine®. All alcohol groups caused an increase in the disintegration force of the elastomeric chains with time.	**LOW**

### Results of individual studies

Type of rinse: Of the authors [8, 11–15, 19] who studied the influence of Listerine® rinse on the degradation of the physical properties of elastomeric chains, Menon et al. [11] found that Listerine®, among the mouthwashes studied, caused the largest percentage of disintegration (71.6%) in a period of 28 days. These results were very similar to those obtained by Ramachandraiah et al. [19], who reported a strength degradation percentage of 69.25%. Other authors 8, 12–14] also found that all groups of alcohol-containing mouthwashes, including Listerine®, showed significant increases in strength degradation compared to the control group (p < 0.01). The authors [8] who studied Cepacol® mouthwash observed a loss of 54.2% at 24 h compared to 53.0% for Listerine®.

Regarding the studies that evaluated mouthwashes with bleaching agents, authors such as Behnaz et al. [17] found a decrease in strength from 48.34 to 53.38%, but without statistical significance (p < 0.05) compared to the control group.

Of the authors [5, 11, 15, 16] who studied mouthwashes with 0.2% chlorhexidine, Omidkhoda et al. [16] found a decrease in strength of 27.24% in the first 24 h compared to non-chlorhexidine mouthwashes, similar to Mirhashemi et al. [4], who observed a significant loss of strength in all groups (p < 0.001). However, in the study by Pithon et al. [15], chlorhexidine did not show a significant influence on the pattern of strength degradation.

In mouthwashes such as Orthokin® or Sensikin® that contain NaF, no effect on the pattern of strength degradation was observed [4]. However, studies [11, 14, 16, 18] that analysed mouthwashes with 0.4%, 0.2%, and 0.05% NaF showed 41–50% losses at 4 weeks.

Another mouthwash analysed was Persica. Omidkhoda et al. [16] reported a strength degradation value ​​of 18.63%, while Mirhashemi et al. [4] reported a loss of 32.4%. Similar results were obtained by Javanmardi and Salehi [4], who obtained higher values ​​of degradation for Persica mouthwash compared to other mouthwashes.

Measurement intervals: Most of the studies included in this review found that the maximum percentage of strength loss occurred at 24 h in all study groups, including the control groups [4, 5, 8, 11, 13, 14, 12, 15, 16, 19]. In the study by Menon et al. [11], this loss was 49.48%, similar to values reported by Al-Ani [13] and Behnaz et al. [17], who observed decreases in strength of 57.85% and 53.38%, respectively.

At 21 days, authors such as Al-Ani [13] observed that the largest percentage of degradation was observed in the Listerine® group, with 57.58% degradation. The lowest degradation values ​​were obtained in the control group (53.65%) and in the Listerine® Zero group (53.82%).

Other studies [11] showed that Listerine® mouthwash also had the highest percentage of decomposition compared to the best results of the Clorhex Plus® mouthwash. In the study by Behnaz et al. [17], lower loss percentages were observed in the control group (64.8%), but the values ​​of the rinses with and without a bleaching agent were very similar, at 66.17% and 66% strength loss, respectively.

Of the time intervals studied, the lowest disintegration percentage was observed at 28 days, and the force was relatively constant. In the study by Behnaz et al. [17], the total degradation value was 86.48% for bleaching agents and 66.3% for control agents. By contrast, in the study by Pithon et al. [15], after 28 days of measurement, the greatest percentage of loss of strength occurred in the control group (distilled water).

### Meta-analysis results

To evaluate the “effect of mouthwash” on the degradation of strength, the difference with respect to the total control group was calculated for each total mouthwash group, combining all studies. To compare the different mouthwashes, any alcohol with a concentration greater than 20% was considered to be of the same type. Similarly, any mouthwash with 0.04% or 0.05% NaF was also considered to be of the same type. Differences were meta-analysed as SMDs.

### Measurement at 24 h

**Fig. 2:**
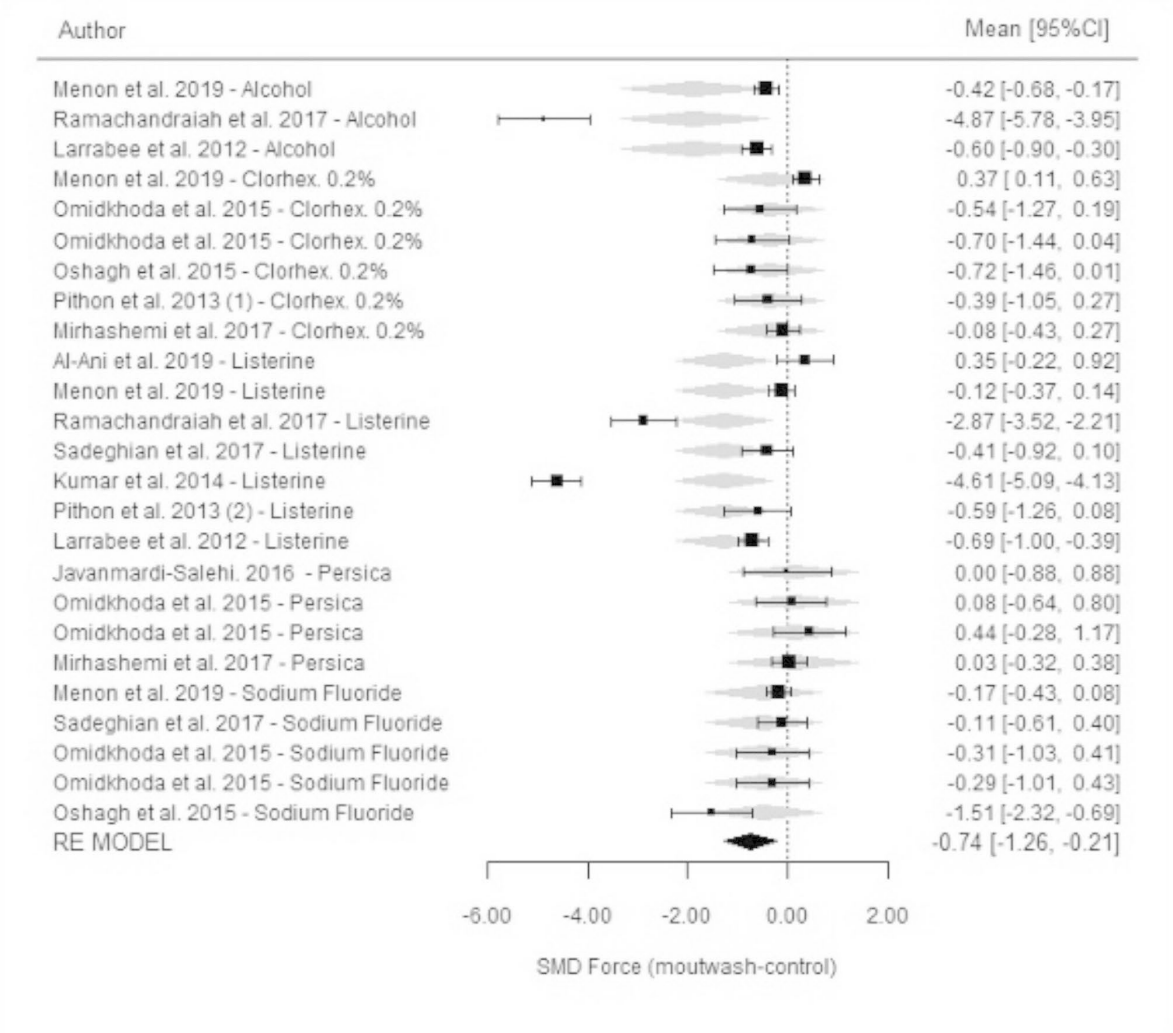
Comparison of force degradation between mouthwashes and the control groups at 24 h for each of the studies included in the meta-analysis. Forest Plot

Regarding the global estimations of the differences between mouthwashes and controls at 24 h, the results showed that, in general, all the mouthwashes were associated with greater force degradation than the control agents (SMD=-0.74; p = 0.006; 95% CI=-1.26, -0.21) (Fig. 2). The heterogeneity of the studies was very high (I^2^ = 97%; p < 0.001) because certain authors reported extreme levels of degradation compared to the control group.

However, no mouthwash studied separately achieved a significant difference with respect to the control group (Persica SMD = 0.10; p = 0.496; 95% CI=-0.18, 0.37; I^2^ = 0%; (Qh) p = 0.789; alcohol 26, 9% SMD= -1.93, p = 0.180, 95% CI-4.75, 0.89, I^2^ = 99.4%, (Qh) p < 0.001; NaF SMD= -0.40, p = 0.063, 95% CI=-0.82, 0.02, I^2^ = 65.1%; (Qh) p = 0.040; chlorhexidine 0.2% SMD= -0.25; p = 0.203; 95% CI-0.64, 0.14; I^2^ = 70.8%; (Qh) p = 0.002)), except Listerine®, which presented a strong tendency, although under a setting of strong heterogeneity (SMD= -1.27; p = 0.059; 95% CI=-1.88, 0.06; I^2^ = 98.5%; (Qh) p < 0.001) .

Regarding the comparison among mouthwashes, no significant differences in the force measured at 24 h (p = 0.200) were observed. In fact, the proximity of the grey diamonds, which symbolize the global effect measurement of the different mouthwashes in Fig. 2, illustrates these similarities.

Although there were no overall differences between the 5 most frequently tested products, interesting trends were observed in the direct comparisons; this was the case for mouthwashes containing alcohol, which had a significantly larger degradation percentage than Persica (p = 0.046) and those containing chlorhexidine 0.2% (p = 0.098). Specifically, Listerine® mouthwash was associated with a larger percentage of degradation than Persica, but statistical significance was not observed (p = 0.085) (Table 3).

**Table 3:** Results of the meta-analysis comparing pairs of mouthwashes at 24 hours, 7 days, 14 days, 21 days and 28 days.

TIME		Alcohol	Chlorhexidine 0.2%	Listerine®	Persica	NaF
24 hours	**Alcohol**					
	**Chlorhexidine 0.2%**	0.098				
	**Listerine**®	0.516	0.194			
	**Persica**	0.046*	0.576	0.085		
	**NaF**	0.144	0.867	0.492	0.492	
7 days	**Alcohol**					
	**Chlorhexidine 0.2%**	0.002**				
	**Listerine**®	0.178	0.024*			
	**Persica**	0.004**	0.936	0.037*		
	**NaF**	0.008**	0.685	0.084	0.658	
14 days	**Alcohol**					
	**Chlorhexidine 0.2%**	0.051				
	**Listerine**®	0.783	0.042*			
	**Persica**	0.064	0.902	0.062		
	**NaF**	0.093	0.835	0.091	0.764	
21 days	**Alcohol**					
	**Chlorhexidine 0.2%**	0.111				
	**Listerine**®	0.941	0.059			
	**Persica**	0.095	0.779	0.058		
	**NaF**	0.156	0.844	0.093	0.651	
28 days	**Alcohol**					
	**Chlorhexidine 0.2%**	0.237				
	**Listerine**	0.585	0.437			
	**Persica**	0.372	0.956	0.565		
	**NaF**	0.546	0.814	0.786	0.813	

Statistically significant = *p < 0,05; **p < 0,01; ***p < 0,001

Regarding the type of connector (short or closed) of the elastomeric chain, no statistically significant differences in strength loss were found among studies at 24 h (p = 0.169; 95% CI=-0.44, 2.50).

### Measurements at 7 days

**Fig. 3:**
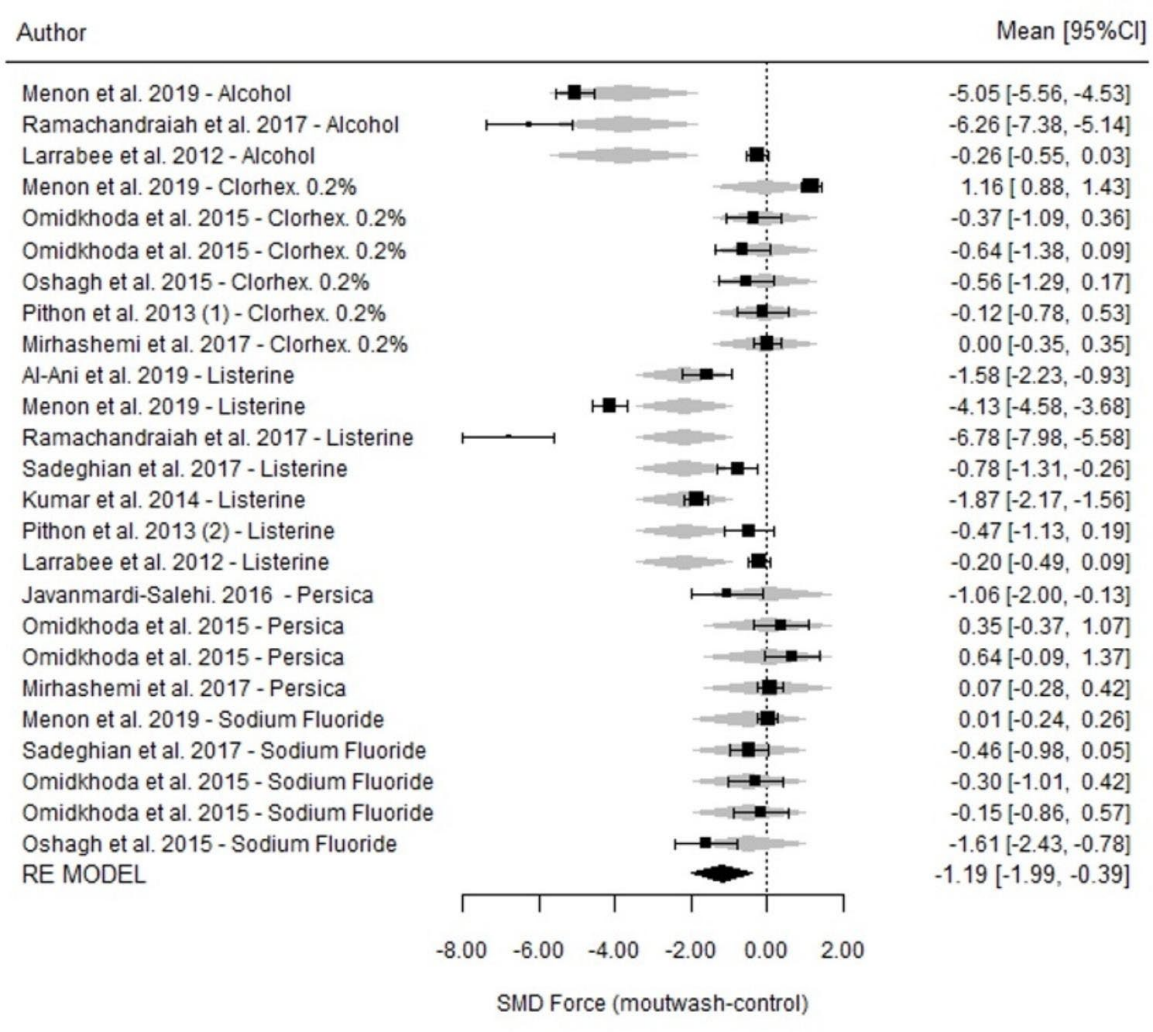
Comparison of force degradation between the mouthwash and control groups at 7 days for each of the studies included in the meta-analysis. Forest Plot

Regarding the overall estimates of the differences between the mouthwashes and controls at 7 days, there were differences among the 5 most frequently used products. In general, all mouthwashes were associated with greater force degradation than the control agents (SMD=-1.19; p = 0.003; 95% CI=-1.99, -0.39) (Fig. 3). The heterogeneity of the studies was very high (I^2^ = 98.6%; p < 0.001) because there were imprecise studies that reported greater degradation, while others reported contrasting data.

Regarding comparisons among mouthwashes, significant differences in the force measured at 7 days were found among mouthwashes (p = 0.005). Mouthwashes containing alcohol had a significantly higher percentage of degradation than those containing chlorhexidine 0.2% and NaF as well as Persica. Alcohol-containing mouthwashes and Listerine® degraded the elastomeric chains in a similar way; the other three mouthwash types also showed similar degradation patterns to one another (Table 3).

Regarding the **type of connector** (short or closed), there were no significant differences in the impacts of the mouthwashes depending on whether they were applied to closed systems or short systems (p = 0.418; 95% CI=-2.65, 1.09).

### Measurements at 14 days

**Fig. 4:**
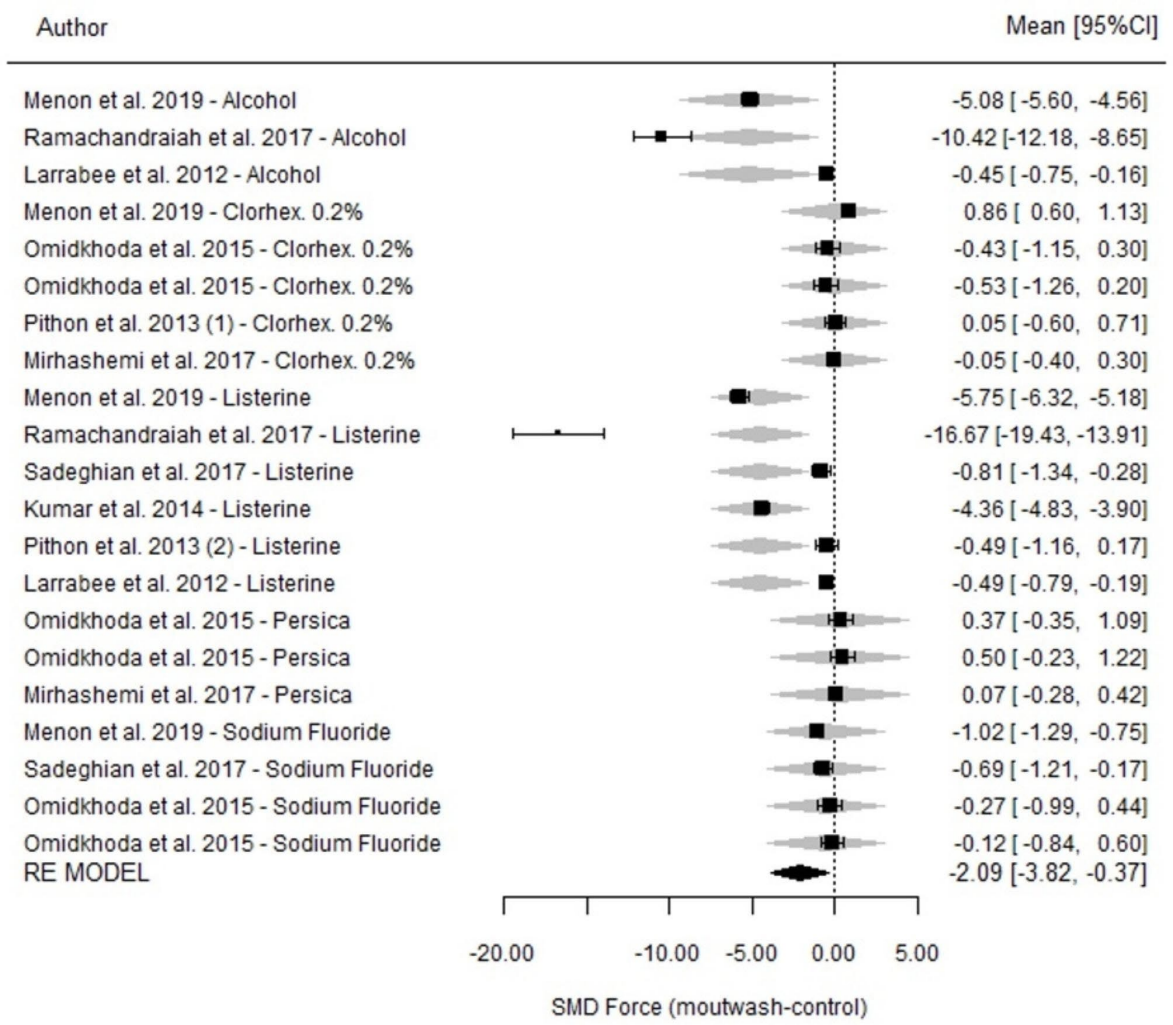
Comparison of force degradation between the mouthwash and its control group at 14 days for each of the studies included in the meta-analysis. Forest Plot

Regarding the differences between mouthwashes and controls, significant differences were interpreted at 14 days (SMD=-2.09; p = 0.018; 95% CI=-3.82-0.36). The experimental products were associated with a significant loss of strength compared to the control agents (Fig. 4). Heterogeneity was high (I^2^ = 99.7%; p < 0.001). After 14 days of measurement, alcohol and NaF showed trends toward loss of strength (the result was significant in the case of NaF) compared to their respective control groups (p = 0.005).

Regarding comparisons among mouthwashes, notable differences were observed, but they were not significant (p = 0.076) (Table 3).

Mouthwashes containing alcohol were associated with a larger percentage of degradation than those containing chlorhexidine 0.2% and NaF as well as Persica.

Regarding the type of connector (short or closed), there were no significant differences in the impacts of mouthwashes applied in closed systems or short systems (p = 0.308; 95% CI=-6.03, 1.90).

### Measurements at 21 days

**Fig. 5:**
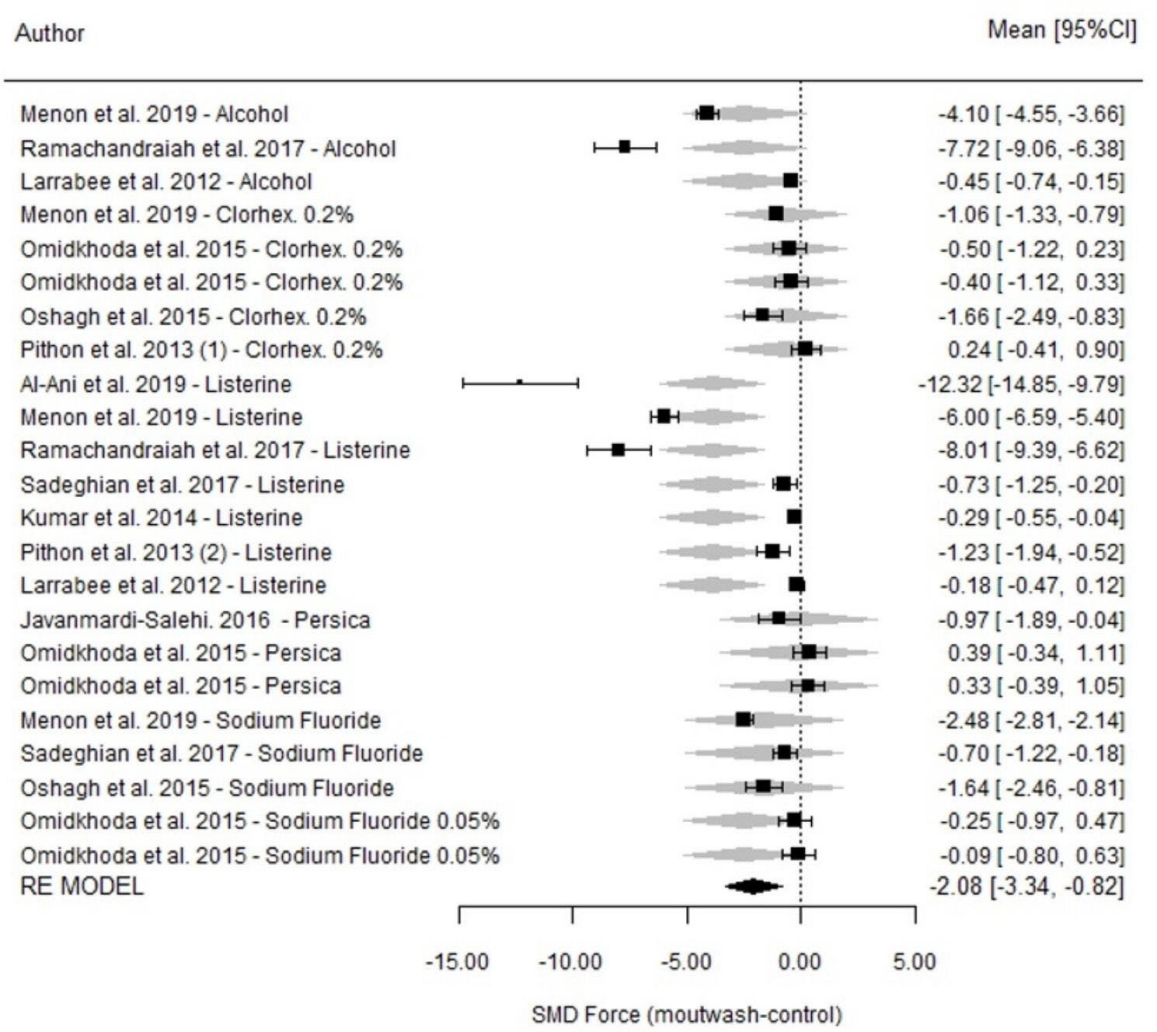
Comparison of force degradation between mouthwashes and their respective control groups at 21 days for each of the studies included in the meta-analysis. Forest Plot

Regarding the difference between the mouthwash and control group, a significant difference was observed (SMD=-2.08; p < 0.001; 95% CI=-3.34, -0.82) (Fig. 5). After 21 days of measurement, Listerine® mouthwash and alcohol-containing mouthwashes were associated with great losses of strength compared to their respective control agents (at the limit of significance). In general, the use of mouthwash was associated with a significant loss in strength compared to use of a control agent (p = 0.001); however, these results were obtained under highly heterogeneous conditions (I^2^ = 99.3%; p < 0.001).

Regarding comparisons among mouthwashes, there were no differences in the strength measured at 21 days among the different mouthwashes (p = 0.120). In general, alcohol-containing mouthwashes, such as Listerine®, degraded the elastomeric chains more than Persica, chlorhexidine 0.2% and NaF (Table 3).

There were no significant differences in the impact of mouthwashes according to closed or short systems (p = 0.183; 95% CI=-4.69, 0.89).

### Measurements at 28 days

**Fig. 6:**
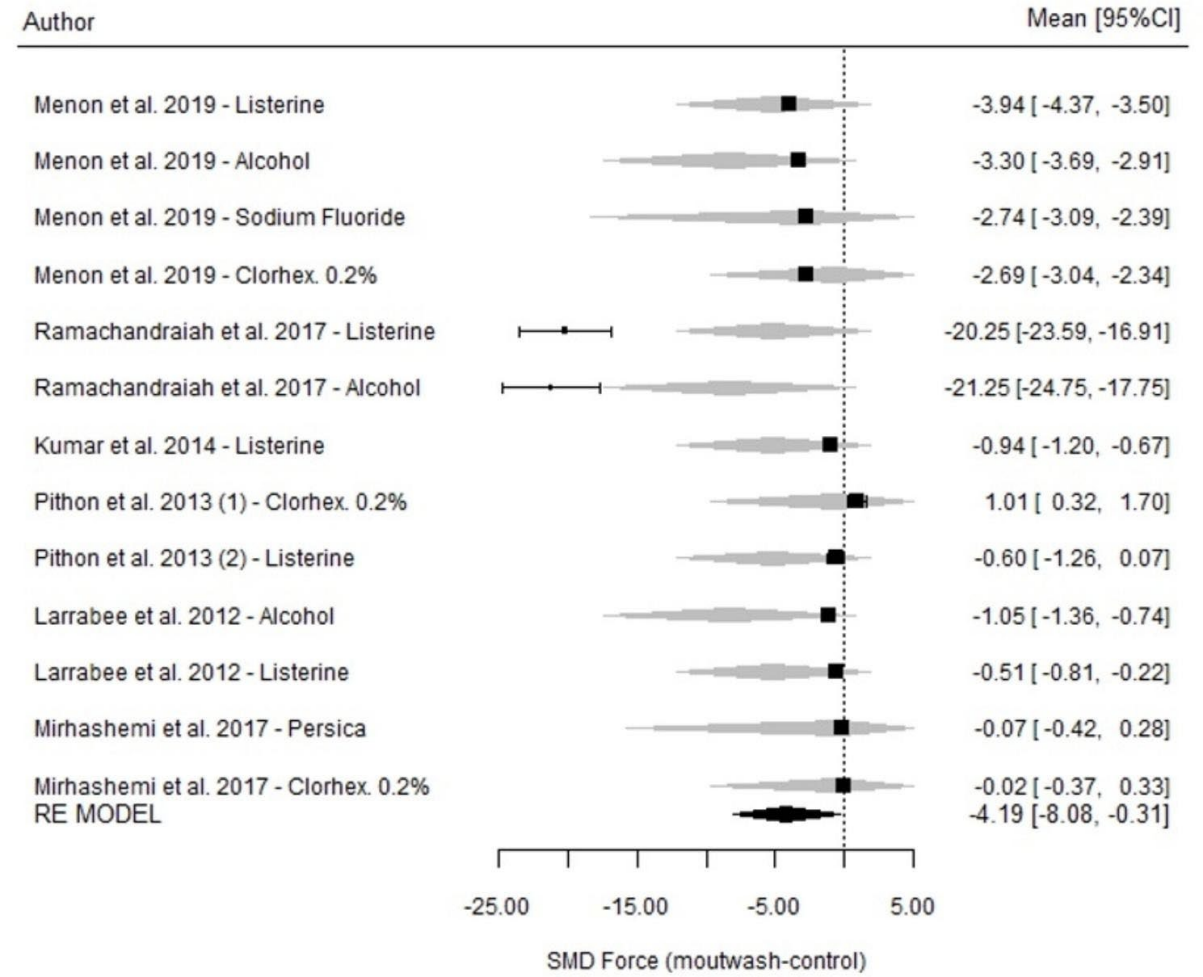
Comparison of force degradation between mouthwashes and their respective control groups at 28 days for each of the studies included in the meta-analysis. Forest Plot

At 28 days of measurement, statistically significant differences were found between the mouthwashes and the control groups (SMD=-4.19; p < 0.035; 95% CI=-8.08, -0.31) (Fig. 6); however, these results were obtained under highly heterogeneous conditions (I^2^ = 99.9%; p < 0.001).

Regarding comparisons among mouthwashes, there were no differences in the strength measured at 28 days between the different mouthwashes (p = 0.778) (Table 3).

There were no significant differences in the impacts of the mouthwashes depending on whether they were applied in closed systems or short systems (p = 0.094; 95% CI=-1.33, 16.9), although there seemed to be a certain tendency towards worse performance in closed systems.

### Risk of bias and quality assessment of individual studies

Table 4 shows the scores of each single parameter analysed (clearly stated aims, explanation of sample size calculation, presence of a control group well defined, operator blinding, number of links used, distance between pins and statistical analysis) and the percentage of quality and risk of bias for the publications included in this systematic review. An overall judgement of a low, medium or high risk of bias (> 70%=low risk of bias, 50–70%=medium risk of bias, and < 50% = high risk of bias) was formed for each study based on the scores from a protocol adapted from the QUIN tool developed by Sheth et al. [10] to systematic reviews of in vitro studies.

**Table 4:** Quality assessment and risk of bias of the included studies. Adequately specified = 2 points, inadequately specified = 1 point, not specified = 0 point, and not applicable = exclude criteria from calculation. Risk of bias (High: <50%; Medium: 50–70%; Low: >70%)

STUDY	Study design	Clearly stated aims	Sample size calculation	Well defined control group	Operator details and blinding	Number links used	Distance between pins	Right statistical analysis	QUALITY RESULTS (%)	RISK OF BIAS
**Al – Ani** [13]	In vitro	2	2	2	0	0	2	1	64,2%	**MEDIUM**
**Menon et al.** [11]	In vitro	2	2	2	1	2	2	2	92,8%	**LOW**
**Behnaz et al.** [17]	In vitro	2	2	2	2	2	2	2	100%	**LOW**
**Nahidh et al.** [20]	In vitro	2	2	2	0	0	2	2	71,4%	**LOW**
**Ramachandraiah et al.** [19]	In vitro	1	2	2	0	2	2	2	78,57%	**LOW**
**Mirhashemi et al.** [4]	In vitro	1	2	2	0	0	2	2	64,2%	**MEDIUM**
**Sadeghian et al.** [14]	In vitro	1	2	2	0	2	2	2	78,57%	**LOW**
**Javanmardi and Salehi** [3]	In vitro	1	2	2	1	2	2	2	85,7%	**LOW**
**Omidkhoda et al.** [16]	In vitro	2	2	2	0	2	2	2	85,7%	**LOW**
**Oshagh et al.** [18]	In vitro	2	0	2	0	2	2	2	71,4%	**LOW**
**Kumar et al.** [12]	In vitro	1	2	2	2	2	2	2	92,8%	**LOW**
**Pithon et al.** [15]	In vitro	2	2	2	0	2	2	2	85,7%	**LOW**
**Pithon et al.** [7]	In vitro	2	2	2	2	2	2	2	100%	**LOW**
**Larrabee et al.** [8]	In vitro	2	2	2	2	2	2	2	100%	**LOW**

In general, twelve studies [3, 7, 8, 11, 12, 14–20] were evaluated as having low risk of bias, with percentages ranging between 71.4% [18, 20] and 100% [7, 17], and only two [4, 13] were considered of medium risk. Al-Ani et al. [13] (with a score of 64.2%) did not specified operator details and blinding, did not quantified the number of links used and did not specified the statistical analysis whereas Mirhashemi et al. [4] (with a score of 64.2%) did not have a well-defined objectives, did not specified operator details and blinding and did not quantified the number of links used.

### Egger’s test (p value) for publication bias

Using Egger’s test, we were able to graphically represent the regression line between the precision of the studies (independent variable) and the standardized effect (dependent variable) and perform a statistical evaluation of bias.

At 24 h, the study by Ramachandraiah et al. [19], assessing 26.9% alcohol, showed strong publication bias (p < 0.001) because the study was more imprecise than those by Menon et al. [11] and Larrabee et al. [8] due to its smaller sample size. The studies by Menon et al. [11], Mirhashemi et al. [4], Oshagh et al. [18], Omidkhoda et al. [16], and Pithon et al. [15], which all assessed 0.2% chlorhexidine, showed clear asymmetry in the funnel plot (p < 0.001). The more imprecise the study was, the more strength degradation was found.

At 7 days, total symmetry was observed among studies assessing some mouthwashes, such as Persica [4, 5, 16], Listerine® [8, 11–15, 19] and NaF-containing mouthwashes [14–16, 18]; however, for studies assessing chlorhexidine-containing mouthwashes, clear asymmetry was observed in the Funnel plot (p < 0.001). The lower the accuracy was, the greater the reported force degradation.

At 14 days, asymmetry was observed; this asymmetry was due to the study by Ramachandraiah et al. [19], who studied Listerine®, since the results were radically different from those in the other studies [8, 11, 12, 14, 15] and the most imprecise.

At 21 days, the studies by Menon et al. [11], Pithon et al. [15] and Omidkhoda et al. [16], who studied chlorhexidine 0.2%, caused asymmetry in the funnel plot (p = 0.089). The observed trend was as follows: the greater the precision of the study was, the clearer the disadvantage of the mouthwash compared to the control group.

At 28 days, the heterogeneity due to the Listerine® [8, 11, 12, 15] studies was very high (I^2^ = 99.9%), and the funnel plot showed that the main cause was the study by Ramachandraiah et al. [19] (p < 0.001). No additional data were available for further comparisons.

## Discussion

Elastomeric chains are auxiliary appliances commonly used in orthodontic treatments for multiple purposes due to their great versatility. However, their efficacy remains a controversial and questionable issue due to the rapid decrease of their mechanical properties and loss of strength over time influenced by different external factors [4–8], 11–20]. Although articles related to the degradation of the strength of elastomeric chains have been published both with in vivo and in vitro, no systematic review with meta-anslysis has been reported [21–23]. In this sense, the daily use of mouthwashes, which are part of routine oral hygiene in orthodontic treatments, can influence the degradation of the strength of the elastomeric chains and, therefore, could promote the loss of effectiveness. In recent years, preprocedural gargling with a mouthrinse, such as chlorhexidine gluconate, was hypothesized to act possibly as an additional protective measure, reducing the microbial oral load of SARS-CoV-2 [6], but at the same time could also have been a key factor contributing to the decrease of the mechanical properties of the elastomeric chains used in orthodontics.

In this review, 14 articles were used for the qualitative and quantitative analysis so the meta-analysis could be performed on all of them. All the studies [3, 7, 8, 11, 12, 14–20], except those of Al- Ani et al. [13] and Mirhashemi et al. [4] were considered to have high quality and low risk of bias. To calculate the quality of the studies, a protocol adapted from the QUIN tool by Sheth et al. [10] was used. Although, many dentistry systematic reviews followed the criteria previously proposed by Sarkis-Onofre et al. [25] in 2014, the authors decided to implement the new proposed tool published in 2022 [10] since they considered it an objective and easily reproducible tool that measures the risk of bias with a formula. Until now, there is no universal criterion or tool that allows evaluating the quality index of in vitro studies in systematic reviews. This has been stated by authors such as Tran et al. [24] who have verified that most quality assessment tools in systematic reviews and meta-analysis of in vitro studies in dentistry were developed by the authors.

When analyzing the results of this meta-analysis, a difference must be made in both the type of mouthwash used and the time of use, since the combination of both factors is key to drawing a conclusion of clinical relevance.

First of all, it should be noted that for all the times analysed, 24 h, 7, 14, 21 and 28 days, the results of this study showed significant differences between the mouthwash groups and the control groups.

In general, the use of mouthwashes during orthodontic treatment, both used punctually or long-term, produces a greater degradation of the strength of the elastomeric chains.

These results were comparable to those reported by other authors [17, 19], who observed statistically significant decreases in strength in all experimental groups with a mean percentage of loss of strength after 24 hours [19] ranging from 41.68 to 55.18% for all the mouthwashes evaluated.

However, during the second week, some authors [11], found that the strength decreased slightly compared to the first week while others [17] obtained very similar results.

It is also remarkable to differentiate between the different types of mouthwashes, since not all of them behave in the same way. The results of the present work did show that there is a greater loss of properties of the elastomeric chains when alcohol-based mouthwashes are used.

Regarding mouthwashes that contain alcohol at different concentrations [8, 11, 15, 19], such as Listerine®, several authors [8, 11–14] obtained similar results to our study with statistical significance. Such studies [8, 11, 19] found that in all groups where an alcohol-agent was used, there was a clear increase in the degradation of the strength of the elastomeric chains in comparison with the control group. When comparing between mouthrinses Menon et al. [11] found a percentage of degradation of 49.48%, in the 26.9% alcohol-based sample while those immersed in chlorhexidine 0.2% showed a smaller percentage of loss (46.7%). Listerine® Total Care Zero group (48.34%) and Listerine® Healthy White with whitening agents group (53.38%) had larger percentages of loss, while the strength loss in the control group decreased by only 42.18%.

As previously seen in the case of Menon et al. [11] the worst degradation occurred in those mouthwashes containing alcohol such as the 26.9% alcohol group, the Listerine® mouthwash group and the Listerine Healthy White® mouthwash group followed by the Listerine® Total Care Zero group.

In our study, for almost all the periods studied (from 24 h to 21 days), 26.9% alcohol-based mouthwashes, such as Listerine®, presented significant differences with respect to the control groups in which artificial saliva was applied. Some authors [12] also found that the greatest loss of strength associated with Listerine® occurred during the first week, and then the strength decreased gradually. Only the study by Al-Ani [13] found that Listerine® mouthwash generated less than a 50.53% decrease in strength versus a 51.61% decrease in the control group for clear chains (Morelli®) after the first week of use. Other authors such as Al-Ani [13] observed that, at 21 days of measurement, the largest percentage of degradation was observed in the Listerine® group, with 57.58% of degradation. The lowest degradation values ​​were obtained in the control group (53.65%) and in the Listerine® Zero group (53.82%). In the study by Behnaz et al. [17], lower percentages were observed in the control group (64.8%), but the values ​​of the rinses with and without a bleaching agent were very similar, with 66.17% and 66% of strength loss, respectively.

The present work found that, only for the period of 28 days, the values were equated and the differences between both groups disappeared. At this time point, neither alcohol-containing mouthwashes (p = 0.184) nor Listerine® mouthwash (p = 0.217) presented significant differences with respect to their controls. Thus, only if the elastomeric chains were changed every 28 days, the affectation of the alcohol-based mouthwashes would be similar to that of the control groups, without there being an increase in the loss of efficacy. Other studies such as the Menon et al. [11] and Ramachandraiah et al. [19] reported a total strength degradation percentage in the Listerine® groups of 71.6% and 69.25% at this time-point.

On the other hand, the results of the present study regarding mouthwashes based on Persica or Chlorxidine 0.2% showed that these two types of mouthwashes behaved similarly to the artificial saliva control group for all the periods studied (from 24 h to 21 days). That is to say, its use does not generate a greater loss of effectiveness of the chains than that suffered by being subjected to artificial saliva.

Of the authors [5, 11, 15, 16] who studied mouthwashes with chlorhexidine 0.2%, Pithon et al. [15], concluded that chlorhexidine did not show a significant influence on the pattern of strength degradation. However, Omidkhoda et al. [16] found a decrease in strength of 27.24% in the first 24 h compared to non-chlorhexidine mouthwashes, similar to Mirhashemi et al. [4], who observed a significant loss of strength in all groups (p < 0.001).

With respect to sodium fluoride-based mouthwashes, the results of this study are somewhat less specific, since significant differences in strength degradation were found with respect to the artificial saliva control groups during the first 24 h, but not during the first week nor at 21 days. And yet, at 14 days, they again showed significant differences.

Finally, the present study also aimed to analyse the influence of the type of link, short or closed, on the resistance of the chains with respect to the use of different mouthwashes. In this case, the effect of mouthwashes was not evident for any of the periods studied, where no significant differences were found according to the type of link used.

Regarding the limitations found when carrying out this review, it should be pointed out that most of the authors provided the exact measurements of recorded force, except Omidkhoda et al. [16] that did it in terms of percentage reduction.

This review found that in most of the studies [4, 5, 7, 8, 11–13, 15–18, 20], only one manufacturer was studied. Only Ramachandraiah et al. [19] and Sadeghian et al. [14] compared three and two products, respectively. Due to the heterogeneity of mouthwashes used in the different studies, it was difficult to compare all of them in the meta-analysis. For example, those containing bleaching agents, were not possible to include in the quantitative analysis.

Apart from that, the literature regarding the distances between links or the importance of the prestreching prior to the activation of the elastomeric chains is scarce with only two articles [16, 20] found that compared the differences between link distances, and only one that compared prestretched and nonprestretched chains [19]. Only Oshagh et al. [18] applied a distance supported by the study of Nattrass et al. [6], who stated that 25 mm was the average distance between the canine and the first molar.

The authors suggest that more studies comparing the effects of the products over different tiem intervals or studies that combine different manufacturers of elastomeric chains with different mouthwashes are needed.

Finally, well designed studies following standardized and strict criteria such as the number of mouthwashes that are measured, the distance between pins and the type of chain used should be performed to obtain more accurate results.

### Conclusion and recommendations

The results of this work showed that a greater force decay of the elastomeric chains is produced after being subjected to the action of different mouthwashes, especially those that contain alcohol. Mouthwashes such as Listerine® and those containing alcohol increase the speed of degradation of the physical properties of orthodontic elastomeric chains. The differences with respect to the control group are greater during the first 21 days, with the values being equated to those of the artificial saliva group after 28 days of use. The results regarding the sodium fluoride-based mouthwashes are controversial, since a greater degradation was observed compared to the control group during the first and third weeks of use. The application of mouthwashes based on Persica or chlorhexidine 0.2% did not produce any adverse effect on the degradation of the chains for any of the periods analyzed.

Thus, clorhexidine 0.2% mouthwashes could be a good alternative due to its low impact on the force decay of elastic chains and its ability to maintain low microbial activity. More in vitro and in vivo studies comparing different manufacturers and other agents such as cetylpyridinium chloride should be performed.

### Electronic supplementary material

Below is the link to the electronic supplementary material.

Supplementary Material 1

## Data Availability

All data generated or analysed during this study are included in this published article [and its supplementary information files].
