# Supplementary material for: Effect of mouthwashes on the force decay of polymeric ligature chains used for dental purposes: a systematic review and meta-analysis
Source: BMC Oral Health. 2023 Aug 4;23:538. doi: 10.1186/s12903-023-03240-3 (PMC10401800; doi:10.1186/s12903-023-03240-3)
Supplement: Supplementary file 1 — Supplementary Material 1 [file 12903_2023_3240_MOESM1_ESM.docx]

**Table S1.** Excluded Articles.

| **AUTHOR** | **YEAR** | **EXCLUSION CRITERIA** |
| --- | --- | --- |
| **Mousavi et al.** | 2020 | Did not address the PICO model. Focused on the degradation of the strength of elastomeric chains but did not assess mouthwashes. |
| **Subramani et al.** | 2020 | Did not address the PICO model. |
| **Triwardhani et al.** | 2020 | Did not address the PICO model. Addressed strength degradation, but did not assess mouthwashes. |
| **Lawal et al.** | 2019 | Did not address the PICO model. Studied ligatures, not chains. Did not assess mouthwashes. |
| **Patel and Thomas.** | 2018 | Did not address the PICO model. Focused on the degradation of force but did not assess mouthwashes. |
| **Rafeeq et al.** | 2017 | Did not address the PICO model. |
| **Losito et al.** | 2014 | Assessed medium-link chains, not short-link chains. |
| **Bratu et al.** | 2013 | Did not address the PICO mode. Studied ligatures, not chains. |
| **Dos Santos et al.** | 2013 | Did not address the PICO model. Did not assess mouthwashes. |
| **Ramazanzadeh et al.** | 2009 | Not performed between 2012-2020. |
| **Eladies et al.** | 2005 | Did not address the PICO model. Assessed single ties, not chains. Did not assess mouthwashes. |
